# Responsiveness of five shoulder outcome measures at follow-ups from 3 to 24 months

**DOI:** 10.1186/s12891-021-04483-3

**Published:** 2021-07-05

**Authors:** Øystein Skare, Jostein Skranes Brox, Cecilie Piene Schrøder, Jens Ivar Brox

**Affiliations:** 1 Surgical Department, Lovisenberg Diaconal Hospital, Oslo, Norway; 2 Orthopedic Department, Lovisenberg Diaconal Hospital, Lovisenberggt 17, 0440 Oslo, Norway; 3 Copenhagen Business School, Copenhagen, Denmark; 4 Department of Physical Medicine and Rehabilitation, Oslo University Hospital, Oslo, Norway; 5 Medical Faculty, University of Oslo, Oslo, Norway

**Keywords:** Minimal important difference, MCID, Responsiveness, SLAP, Shoulder, EQ-5D3L, Clinical scores, PROMS

## Abstract

**Background:**

To assess responsiveness of five outcome measures at four different follow-ups in patients with SLAP II lesions of the shoulder.

**Methods:**

119 patients with symptoms and signs, MRI arthrography and arthroscopic findings were included. The Western Ontario Shoulder Instability Index (WOSI), Oxford Instability Shoulder Score (OISS), EuroQol (EQ-5D3L), Rowe Score and Constant-Murley Score (CMS) were assessed at baseline, 3, 6, 12 and 24 months. The analysis contains both anchor-based and distribution-based methods, and hypothesis testing.

**Results:**

Confidence intervals for ROC cut-off values, representing MID, for OISS, CMS and EQ-5D3L crossed zero at 3 months. Cut-off values were stable between 6- and 24-months follow-up. At 24-months ROC cut-off values (95% CI) were: Rowe 18 (13 to 24); WOSI 331 (289 to 442); OISS 9 (5 to 14); CMS 11 (9 to 15) and EQ-5D3L 0.123 (0.035 to 0.222). MID_95%limit_ estimates were substantially higher than ROC cut-off values and MID_MEAN_ at all follow-ups for all instruments. The reliable change proportion (RCP) values in the improved group were highest for WOSI and the Rowe Score (ranging from 68 to 87%) and significantly lower for CMS. EQ-5D3L had the lowest values (13 to 16%). We found a moderate correlation between mean change scores of the outcome measures and the anchor, except for the EQ-5D3L.

**Conclusions:**

In patients with SLAP II-lesions the patient reported OISS and WOSI and the clinical Rowe score had best responsiveness. Our results suggest that 3 months follow-up is too early for outcome evaluation.

## Background

Clinical scores, and patient reported outcome measures (PROMS) are recommended for evaluation of treatment effects in patients with shoulder disorders. Over the last decades, the patient perspective has been considered increasingly important [1] and shoulder specific and generic health related quality of life outcome measures may replace clinical scores [2]. The shoulder specific questionnaires commonly include questions about pain and activities in daily life. The questionnaires are developed for shoulder patients in general or for specific subgroups, for example patients with instability. The answers are transformed into metrics and the scientific field handling the corresponding issues are labelled psychometrics or clinimetrics [3, 4]. Researchers and clinicians should keep in mind that some information is lost when pain and disability are transformed into metrics and that responsiveness is a word used to describe sports cars [5]. Clinimetric responsiveness describes the ability of an outcome measure to detect a change that is not at random.

Generic quality of life questionnaires, like the EuroQol (EQ-5D3L), are often applied as an utility index in cost effectiveness studies [6], despite the fact that its reliability and usefulness in shoulder patients have been questioned [7]. While a possible advantage of questionnaires is that they may be answered online to save time and costs of consultations and travel, the clinical scores provide additional information about range of motion, muscle strength and stability that may be important for the clinician and the patient. However, observations are prone to blinding and inter- and intra-rater measurement error.

An outcome measure in patients with a shoulder disorder should be evaluated for reliability and validity. Responsiveness refers to the validity of change scores while criterion validity refers to the validity of a single score. Hypothesis testing is recommended to assess validity. Hypotheses should be predefined and assess the direction and difference in change. The minimal important difference (MID) provides the clinician with an estimate of the difference between no change and minimal change on the outcome measures according to patient perceived improvement and is commonly used as a measure of responsiveness. The MID is different from a moderate or large treatment effect and the proportion of patients above MID in a study is not equivalent with the success rate. There are several methodological concerns, for example: What is the best follow-up time for a valid assessment of MID? Are the measures of responsiveness different for clinical scores and PROMS? How should MID be estimated, and how should uncertainty both in measurement and in methodology be considered?

There are no outcome measures developed specifically for patients treated for SLAP- lesions [8]. The 1998 version of the Rowe Score, WOSI, OISS and EQ-5D3L have previously been validated at 6 months [7–9]. The CMS is a recommended clinical score for all types of shoulder disorders but has not been validated for use in patients with SLAP lesions [10]. The aim of the present study was to compare the responsiveness of these five different outcome measures at four different follow-ups (3, 6, 12 and 24 months) in patients with SLAP II lesions using both anchor- and distribution-based methods. The second aim was to evaluate validity of the outcome measures by hypothesis testing.

## Methods

### Study design and settings

This is a prospective methodology study combining the use of distribution- and anchor-based methods, and hypotheses to assess responsiveness of outcome measures in patients with type II SLAP lesions from 3 to 24 months follow-up.

Patients were recruited from the outpatient clinic at the Department of Orthopaedic Surgery at Lovisenberg Diaconal Hospital between January 2008 and January 2014. They were originally enrolled in a blinded, three-armed, randomized, sham-controlled study with a 24 months follow-up assessing the clinical effectiveness of arthroscopic labral repair and biceps-tendinosis in patients with type II SLAP lesions [11, 12]. Some of these patients were included in a study of responsiveness at 6 months follow-up [9]. The patients enrolled were between 18 and 60 years old and had experienced shoulder pain and disability for at least 3 months prior to inclusion, despite having received non-operative treatment (physical therapy, non-steroid anti-inflammatory drugs, corticosteroid injections). They had symptoms, clinical findings and MR –arthrography indicating type II SLAP lesion. The diagnosis was confirmed during arthroscopy. The inclusion- and exclusion criteria, and a flow chart, have been described in detail previously [11, 13]. We obtained written informed consent from all participants. Ethics approval (IRB00001870) was received from the Ethics Committee Health Region Southeast, Oslo, Norway. The protocol was registered at ClinicalTrials.gov (NCT00586742).

Patients enrolled in the study completed the WOSI, OISS and EQ-5D3L at baseline and at 3, 6, 12- and 24-months follow-up. Comparisons between groups have been published previously [11]. The clinical Rowe score (1988-version) and CMS were completed by one single experienced Manual Therapist (ØS) at all follow-ups.

### Outcome measures

We used previously translated and validated Norwegian versions of the patient reported WOSI [14], OISS [15] and EQ-5D3L [7, 16]. All outcome measures including the Rowe score and CMS are described in Table 1 [17, 18].

**Table 1:** Outcome measures

Outcome measure	Domains	Score
*Patient rated outcome measures*		
WOSI*	21 questions answered on a continuous scale from 0 to 100 covering physical symptoms; sports; recreation and work; lifestyle; and emotions	0 to 2100 (worst possible condition)
OISS*	12 questions with five response alternatives (5 to 0 points) covering instability, daily activities, pain, work, social life, sports/hobbies, attention to the shoulder problem, lifting and lying position	12 to 60 (worst possible function)
**EQ-5D**	5 questions with three response categories covering mobility; self-care; usual activities; pain/discomfort; and anxiety/depression	−0.59 to 1.0 (best quality of life)
*Clinician evaluated outcome measures*		
**Rowe score#**	5 domains evaluated: pain; stability; function, range of motion measured by a goniometer; and muscle strength using a spring gauge. Pain (25 points) and function were reported on five-step categorical scales.	0 to 100 (best possible state)
**Constant-Murley Score**	4 domains evaluated: pain intensity; activities of daily living (sleep, work, recreation and hand positioning); active range of motion measured by goniometer and strength of abduction measured with a spring gauge.	0 to 100 (best possible shoulder function)

* For statistical analyses, WOSI and OISS scores were inverted for easier comparisons with the other outcome measures

# At the follow-up examinations, patients were told to rate the state of their shoulder as excellent, good, fair or poor (Rowe Patient Evaluation)

### Anchor for important change

**Fig. 1:**
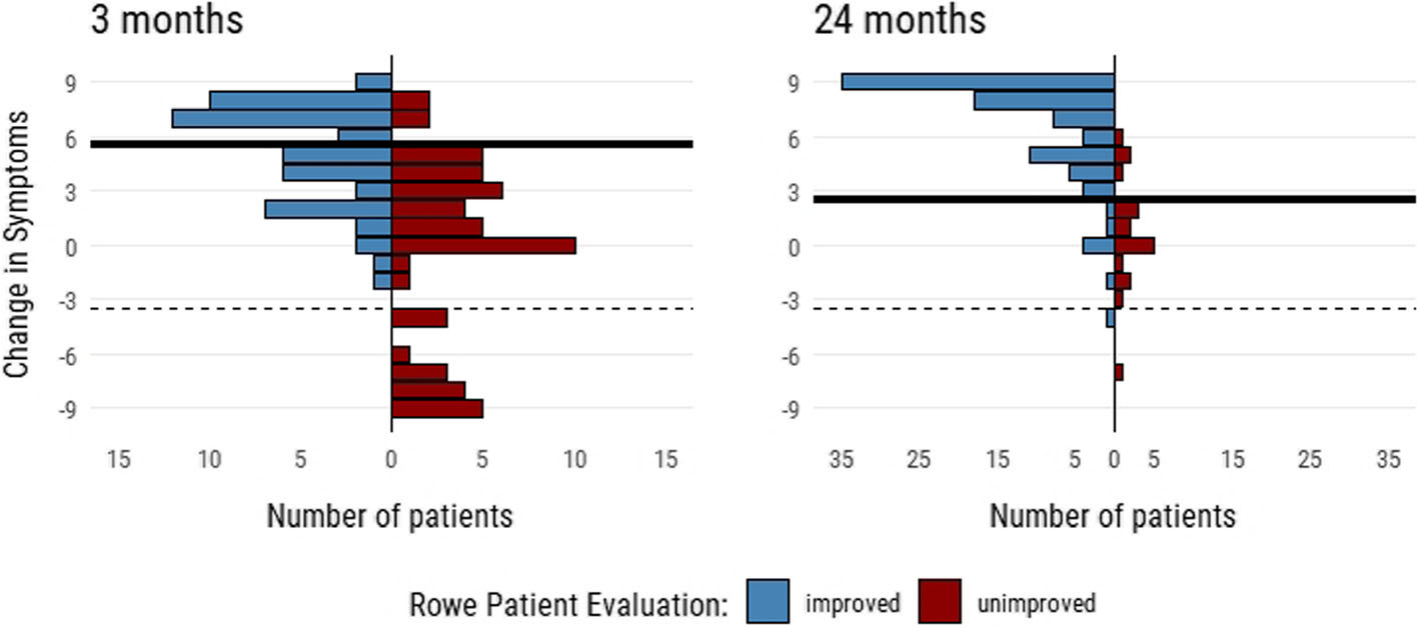
Distribution of Change in Symptoms grouped by improved/unimproved at 3- and 24-months follow-up. Improved/unimproved is defined by Rowe Patient Evaluation were patients responding “Excellent” or “Good” are considered improved while patients responding “Fair” or “Poor” are considered unimproved. The solid black line marks the cut-off point for improved/unchanged. The dotted black line marks the cut-off between unchanged/deteriorated

The anchor was an assessment of change of symptoms on a continuous scale ranging from - 9 (worst possible change of symptoms) to 9 (best possible) and Rowe patient evaluation [19]. This is a question were patients state their shoulder as “Excellent”, “Good”, “Fair” or “Poor”. To divide patients into groups of improved/unchanged/deteriorated both questions were combined using distribution plots. This is further described in the statistics section and in Fig. 1.

### Sample size

Sample size was calculated to accommodate the RCT and was above the general recommendations for estimation of MID [11, 20, 21].

#### Statistics

Total scores for all outcome measures were calculated at all follow-ups. For WOSI missing values were imputed if one or two questions were missing, using the mean value within each subcategory for the given patient. For CMS and OISS we imputed the mean value of the given question. All together 13 observations were imputed.

### Responsiveness

Responsiveness was calculated and investigated using SRM (Standardized Response Mean), RCP (Reliable Change Proportion) and Receiver operating characteristic (ROC) at all follow-ups. The improved and unchanged group were defined by the anchor described above. Cut-off values were decided using distribution plots of the change of symptoms anchor grouped by a dichotomized version of Rowe Patient Evaluation (Fig. 1). This yielded cut-off values on the change of symptoms anchor of 6, 4, 3 and 3 at the different follow-ups. Patients scoring below − 3 were considered deteriorated and excluded from the analysis [22]. Patients with a score between − 3 and cut-off were considered unchanged. We chose not to estimate MID for the deteriorated group due to the small sample size (ranging from 2 to 16).

SRM was estimated by dividing the MCS (Mean Change Score) by the standard deviation of the MCS. 95% confidence intervals were obtained by non-parametric bootstrap estimation. Confidence intervals for baseline scores and MCS were estimated using the normal distribution. All confidence intervals for EQ-5D3L were obtained by non-parametric bootstrap estimation due to non-normal distributions.

RCP was defined as the percentage of patients improved by more than the MDC (Minimal Detectable Change). MDC estimates for all outcome measures, except CMS, were obtained from an earlier study using partially the same sample [7, 8]. MDC for the CMS was estimated by averaging the findings of earlier studies [23–26]. A robustness check for the CMS was performed using the lowest published MDC we could find [23]. 95% confidence intervals were estimated using the Clopper-Pearson method [27].

ROC analysis was incorporated to assess each instrument’s ability to correctly classify patients as improved or unchanged. The sensitivity is defined as the proportion of improved patients correctly classified as improved, while the specificity is the proportion of unchanged patients correctly classified as unchanged. The ROC graph is a plot of the sensitivity against 1-specificity, illustrating the trade-off between false positives and true positives at all thresholds [28]. The optimal threshold was selected at the point on the ROC curve that minimizes the sum of squares of 1-sensitivity and 1-specificity, or equivalently the point closest to the upper-left corner [29].

### Minimal important difference

MID is defined and estimated in a variety of ways in the literature, and there is a lack of formal agreement on which methods are superior [2]. This study incorporates anchor-based distribution methods (SRM, RCP, MID_MEAN_) and ROC analysis (ROC cut-off, MID_95%limit_). ROC cut-off (often referred to as MID_ROC_) is defined as the optimal threshold retrieved from ROC analysis [22, 30, 31]. 95% CI was estimated using a stratified bootstrap procedure, keeping the proportion of improved/ unchanged patients constant in each replicate sample. 95% CI for ROC_AUC_ was estimated using the DeLong method [32].

MID_MEAN_ was defined as the MCS of the patients scoring slightly above the chosen cut-off value on the anchor (i.e. for 6 months this equals patients scoring 4 and 5). The idea is that this group of patients consider themselves minimally improved, and their MCS can therefore be used to identify a MID estimate [30].

MID_95%limit_ was calculated as μ_*change*_ + 1.645 · σ_*change*_ of the unchanged group. This corresponds to the 95% upper limit of the distributions of patients not experiencing an important improvement, and is equivalent to the cut-off value at the 95% specificity on the ROC curve [22]. Statistical analyses were performed in R, version 3.6.2 [33]. ROC analysis was performed using the pROC-package [34].

### Hypotheses

Hypotheses were defined to further evaluate the responsiveness and validity of the instruments and anchor. The following null hypotheses were formulated for all instruments at all follow-ups if not otherwise stated:

1. MCS for men and women are equal.

2. MCS for patients above and below 40 years are equal.

3. The correlation between the MCS and change in symptoms (− 9 to 9) ≥ 0.70.

4. The correlation between the MCS and the anchor (improved/unchanged) ≥ 0.50.

5. MCS for patients with postoperative stiffness is ≤ the MCS for patients without postoperative stiffness at both 3- and at 6-months follow-up.

6. The correlation of the MCS between the instruments ≥0.70.

Hypotheses (1), (2) and (5) were tested using an independent sample t-test or Wilcoxon rank-sum test. Postoperative stiffness was defined as a loss of passive (glenohumeral) range of motion of > 30° in external rotation and abduction. Hypotheses (3) and (4) were tested using Spearman’s rank correlation. Hypothesis (6) was tested using Pearson’s r correlation. A correlation was defined as high > 0.70, as moderate between 0.40 and 0.70, and low < 0.40.

## Results

119 patients were included (Table 2). Eight, four, six and six patients, respectively, did not answer the change in symptoms question at the different follow-ups and were excluded from the analysis. Sixteen (14%), 12 (10%), six (5%) and two (2%) patients answered below − 3 on the anchor at the different follow-ups and were considered deteriorated. The unchanged/improved ratio at 3, 6, 12- and 24-months follow-up was 64/31, 32/71, 17/90 and 21/90.

**Table 2:** Descriptive statistics

Males/Females	72/47
Age (years), mean (range)	40.1 (18–64)
Duration of symptoms (months), median (range)	26 (6–360)
Dominant shoulder	89 (75)
Manual labour	47 (39)
Postoperative capsular stiffness	
3 months	30 (25)
6 months	17 (14)
Physical activity	
None	50 (42)
Weekly	61 (51)
Competition	7 (6)
Treatment	
Sham surgery	40 (34)
Biceps tenodesis	39 (33)
Labral repair	40 (34)

The number (%) of patients is given if not otherwise stated

Baseline total score, MCS, SRM and RCP with 95% CI grouped by the anchor are reported in Table 3. In the improved group the SRM values for Rowe and WOSI were significantly higher than for the CMS and EQ-5D3L at all follow-ups, and for OISS at 3 months follow-up. The CMS was significantly higher than EQ-5D3L at 12- and 24-months follow-up. SRM values for EQ-5D3L ranged from 0.67 to 1.05 (moderate to high). All other SRM values were considered high in the improved group. In the unchanged group all SRM values were low at 3 months follow-up, ranged from low to moderate at 6- and 12-months follow-up, and moderate to high at 24 months follow-up.

**Table 3:**
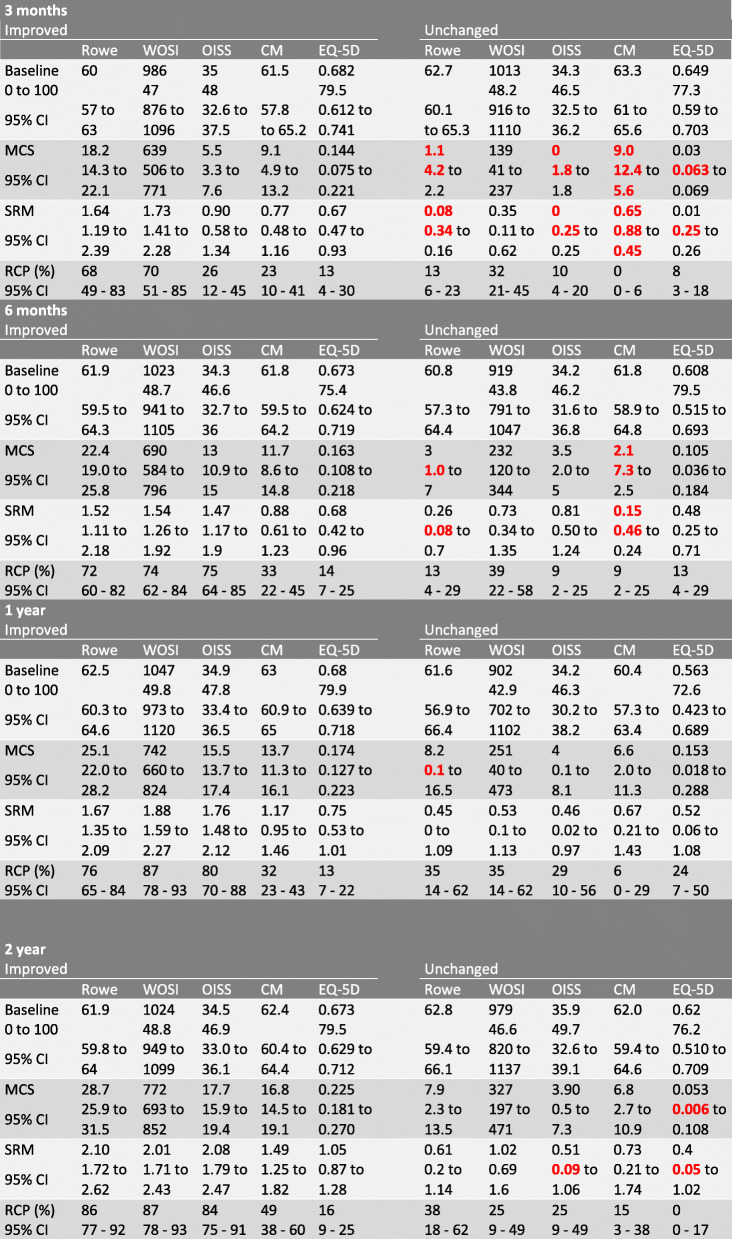
Distribution based responsiveness in improved and unchanged patients

Numbers in bold/red are negative values. *Baseline*, mean total score at baseline; *0 to 100,* baseline score transformed from original scale to a 0 to 100 scale; *MCS*, mean change score from follow-up to baseline; *SRM*, mean change score divided by the standard deviation of the mean change score; *RCP*, proportion of patients with change score exciding the Minimal Detectable Change

RCP values in the improved group were highest for Rowe and WOSI at all follow-ups (ranging from 68 to 87%), with similar values for OISS at 6, 12- and 24-months. EQ-5D3L had the lowest values, and contrary to the other instruments, there was no increase over time (ranging from 13 to 16%). RCP values for the CMS ranged from 23 to 49%, using 16 as the MDC. When using 12 as the MDC the RCP values for the improved group were 32, 43, 56 and 69%.

### ROC analysis and MID

**Fig. 2:**
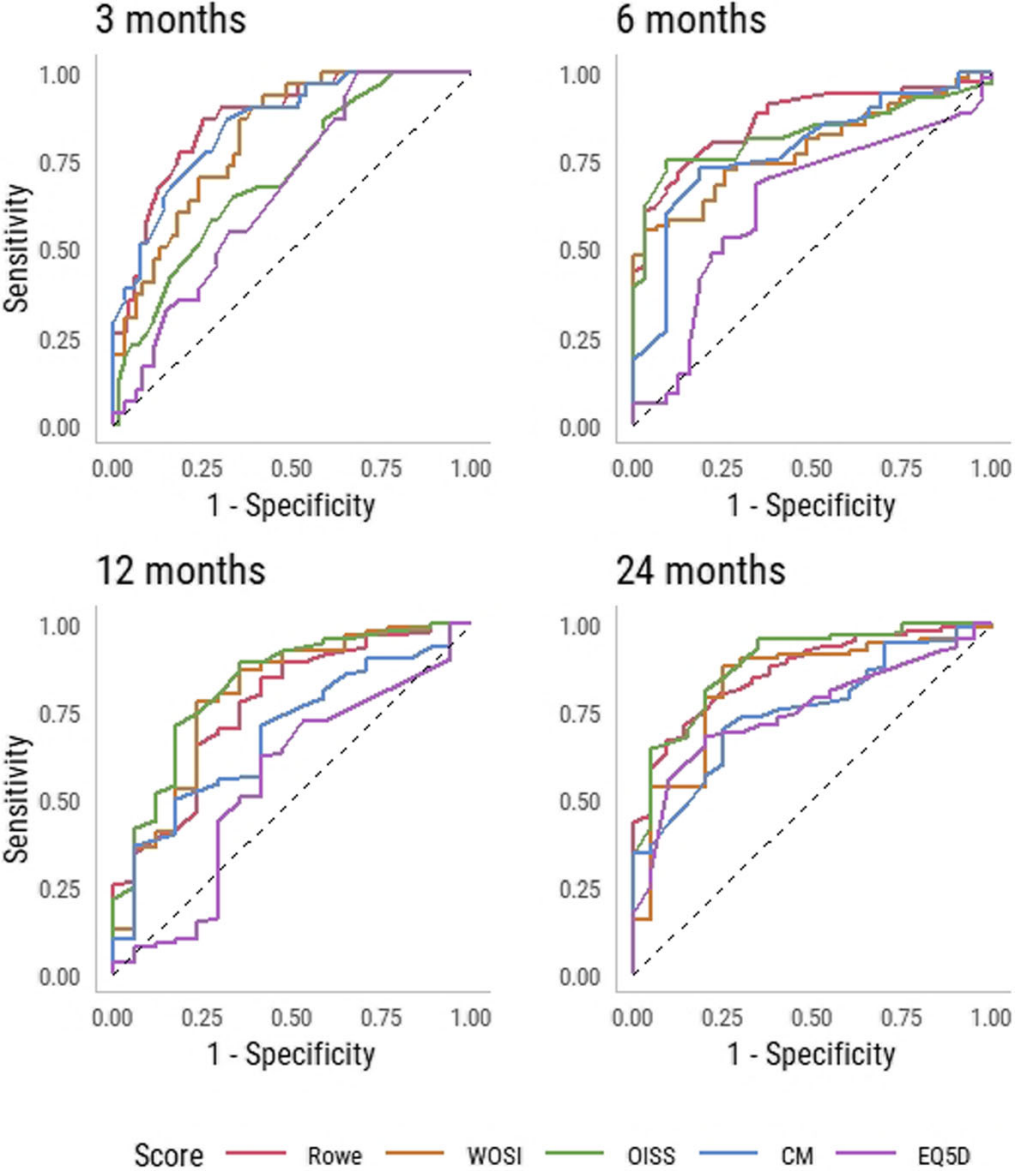
ROC curves of all instruments at all follow-ups

Fifteen out of twenty ROC_AUC_ values were > 0.70 (Table 4). EQ-5D3L had the lowest values ranging from 0.55 to 0.74. ROC curves for all instruments at all follow-ups are reported in Fig. 2. Cut-off values were low for all scores at 3 months follow-up. The OISS, CMS and EQ-5D3L had confidence intervals crossing zero. At 6 months follow-up all cut-off values increased substantially, and were stable between 6- and 24-months follow-up. At 24 months follow-up ROC cut-off values (0 to 100 scale) was 18 for Rowe, 331 (16) for WOSI, 9 (19) for OISS, 11 for CMS and 0.123 (45) for EQ-5D3L. Excluding EQ-5D3L the largest difference between the instruments was 8 on a 0 to 100 scale.

**Table 4:**
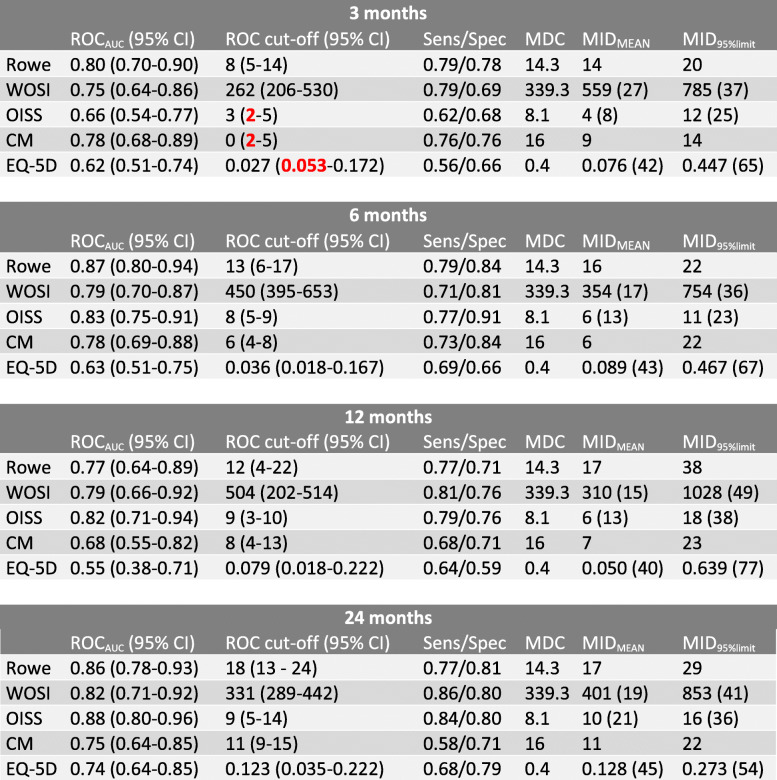
Minimal important difference

Numbers in bold/red are negative values. *ROC*_*AUC*_, area under the ROC curve; *ROC cut-off*, change score threshold that minimizes the sum of squares of 1-sensitivity and 1-specificity (i.e. the point on the ROC curve closest to the upper-left corner); *Sens/Spec*, proportion of improved patients correctly classified as improved/proportion of unchanged patients correctly classified as unchanged; *MDC*, Minimal Detectable Change; *MID*_*MEAN*_, mean change score of patients scoring slightly above the chosen cut-off value on the anchor (0 to 100 scale); *MID*_*95%limit*_, 95% upper limit of the change score distribution of patients defined as unchanged (0 to 100 scale)

MID_95%limit_ estimates were substantially higher than ROC cut-off values at all follow-ups for all instruments. The estimates peaked at 12 months follow-up for all instruments, while being comparable at the other follow-ups. At 24 months follow-up MID_95%limit_ values (0 to 100 scale) were 29 for Rowe, 853 (41) for WOSI, 16 (36) for OISS, 22 for CMS and 0.273 (54) for EQ-5D3L.

MID_MEAN_ values were higher than ROC cut-off values, but lower than MID_95%limit_ values, at 3 months follow-up for all instruments. At all other follow-ups MID_MEAN_ and ROC cut-off values were comparable. MID_MEAN_ values (0 to 100 scale) at 24 months follow-ups were 17 for Rowe, 401 (19) for WOSI, 10 (21) for OISS, 11 for CMS and 0.128 (45) for EQ-5D3L.

At 24 months follow-up MID estimates were lower than the MDC for CMS (except MID_95%limit_) and EQ-5D3L. For the other instruments all MID estimates were higher than the MDC (ROC cut-off value for WOSI was approximately equal).

### Hypothesis testing

There was no evidence of any difference in mean change score between males and females or between patients aged below or above 40 years for any instrument at any follow-up (H1 and H2). All correlations between the MCS and the change in symptoms question were positive (the lowest being 0.33), but only two were > 0.70 (OISS at 6- and 12-months follow-up). The correlations ranged from 0.58 to 0.62 for Rowe, 0.55 to 0.69 for WOSI, 0.47 to 0.74 for OISS, 0.52 to 0.60 for CMS and 0.33 to 0.46 for EQ-5D. Correlations were lowest at 3 months follow-up (H3). Nine of sixteen correlations between the MCS and the anchor (improved/unchanged) for all instruments except EQ-5D were > 0.50. Correlations ranged from 0.43 to 0.60 for Rowe, 0.44 to 0.55 for WOSI, 0.40 to 0.59 for OISS, 0.38 to 0.56 for CMS and 0.13 to 0.32 for EQ-5D (H4). The MCS for patients with postoperative stiffness were significantly smaller than for patients without postoperative stiffness at 3 months follow-up for all instruments. At 6 months follow-up the null hypothesis could only be formally rejected for the Rowe score and the CMS (the *p*-value for OISS and WOSI was 0.05 and 0.06, respectively) (H5). The correlation of the MCS among the instruments at the different follow-ups ranged from 0.58 to 0.71 for Rowe/WOSI; 0.62 to 0.70 for Rowe/OISS; 0.74 to 0.81 for Rowe/CMS; 0.35 to 0.58 for Rowe/EQ-5D; 0.69 to 0.83 for WOSI/OISS; 0.59 to 0.71 for WOSI/CMS; 0.48 to 0.57 for WOSI/EQ-5D; 0.59 to 0.64 for OISS/CMS; 0.45 to 0.48 for OISS/EQ-5D and 0.35 to 0.48 for CMS/EQ-5D (H6).

## Discussion

This study has evaluated responsiveness in five different outcome measures at four follow-ups. The MID estimates derived from the ROC analysis should be interpreted along with other estimates, particularly the mean change score and the measurement error of the instruments.

Estimates obtained at 3 months were not interpreted as meaningful for measuring outcome, particularly the confidence intervals for ROC cut-off values crossed zero for the OISS, CMS and the EQ-5D3L. This may reflect the short time period after surgery with large variability in the improvement process. Few patients had improved and some were deteriorated because of complications like stiff shoulders. Many patients had not regained their muscle strength. These factors influenced the scoring of outcome questions*.* A previous study evaluating patients with rotator cuff tears reported a MID of 10.4 at 3 months [35]. They did not report 95% CI and the ROC cut-off value was 2, which question their findings. We do not recommend the follow-up at 3 months after surgery for estimation of MID.

The distribution-based methods indicate that the CMS is less sensitive to change compared to OISS, WOSI and the Rowe score. MCS, SRM and RCP values were lower for CMS at all follow-ups. A different sensitivity to change was shown for the two clinical scores although baseline values for the improved group were similar. At 2 years the mean score for the CMS was 79.2 in the improved group, while it was 90.6 for the Rowe score. This indicates that for these patients the clinical scores do not scale equally. An increase of one point on the CMS equals a greater improvement on the Rowe score. This might also explain why all MID estimates are lower for CMS than for the Rowe score and suggests that low MID values should not automatically be interpreted as better than higher ones.

### Estimates of minimal important difference

The MID_95%limit_ estimates were substantially higher than the other MID estimates for all outcome measures. As de Vet et al. points out a challenging question is which cut-off point to prefer [22]. A factor driving the high MID_95%limit_ values was the high variation in change score among the unchanged patients. This possibly reflects that other health related issues affect their change score, or difficulties with the anchor in identifying patients who are minimally improved. Because every point on the ROC curve represents a trade-off between sensitivity and specificity, increasing the specificity to 0.95 comes at a cost. By example, at the 24 months follow-up the MID_95%limit_ for the Rowe score was 29 while the cut-off value when maximizing both was 18. The sensitivity and specificity were 0.77 and 0.81 for the latter (Table 4) and 0.56 and 0.95 for the former. We found no reason to dislike false negatives more than false positives, and therefore preferred the ROC cut-off value over the MID_95%limit_.

MID_MEAN_ is a less common way to measure the minimal clinically important change utilizing the fact that a continuous variable identifying the change in main complaint was collected at all follow-ups (− 9 to 9 scale). The disadvantage is related to identifying the group that one considers minimally improved. For example, at 6 months we defined patients scoring 4 and 5 as minimally improved and measured MID_MEAN_ as the mean change score among these patients. Alternatively, we could have used the median or the first or the third quartile emphasizing either *important* or *minimal* change. MID_MEAN_ values were comparable to ROC cut-off values at 6, 12- and 24-months follow-up.

At 24 months follow-up all MID estimates were lower than the MDC for the CMS and EQ-5D3L. In agreement with a recent systematic review we consider MID values that are lower than the measurement error (MDC) as problematic [2]. Recent studies present MID values that are below the MDC without any further discussion [2]. A disadvantage of the present study is that we have not provided MDC estimates for the CMS calculated in this sample. However, we conducted a robustness check using the lowest estimate published and the average MDC values from other studies.

The responsiveness of the EQ-5D3L was inferior to the other outcome measures. We consider this observation to be important because EQ-5D3L was recently used as an utility index in a systematic review to evaluate cost-effectiveness [6].

### Hypothesis testing

We found a moderate correlation between mean change scores of the outcome measures and the anchor, except for the EQ-5D3L [36]. Low correlations between EQ-5D3L and the anchor are also illustrated by low ROC_AUC_ values, and the outcome measure is not suitable to detect improvements in this population.

### Strengths and limitations

The strengths of this study include the use of both clinical scores and PROMS, at four different follow-ups. All clinical assessments were conducted by one single experienced assessor. Comprehensive statistical analyses were conducted for each outcome measure at all follow-ups. Estimates of MID were compared with estimates of MDC. The main challenge was identifying a valid anchor [1]. We found that some patients answered the anchor inconsistently, which may relate to the questionnaire or the heterogeneity of the patients. Some patients had complaints mainly related to specific sports, while others had daily pain and disability related to ordinary activities. Also, recall bias influence the anchor. A previous study found that global perceived change was influenced by the patients’ state at the time of asking [37].

We recommend to use the MCS of the instruments as the primary outcome in trials rather than the proportion of patients exceeding the MID. On the other hand, MID values are helpful in calculating sample size and for understanding results in clinical practice but should be interpreted with an understanding of uncertainty and measurement error of the Instrument [7, 8, 38], the patient group and follow-up time examined.

## Conclusions

In patients with SLAP II-lesions the patient reported OISS and WOSI and the clinical Rowe score had best responsiveness. Our results suggest that 3 months follow-up is too early for outcome evaluation. EQ-5D3L did not have appropriate measurement properties to assess responsiveness in this patient group.

## Data Availability

The datasets generated and analyzed during the current study are not publicly available due to lack of patient consent and recommendation from University of Oslo. However, data can be provided on request to the corresponding author: oystein.skare@lds.no
